# Risk of Readmission for Wheezing during Infancy in Children with Congenital Diaphragmatic Hernia

**DOI:** 10.1371/journal.pone.0155556

**Published:** 2016-05-12

**Authors:** Gregoire Benoist, Mostafa Mokhtari, Antoine Deschildre, Naziha Khen-Dunlop, Laurent Storme, Alexandra Benachi, Christophe Delacourt

**Affiliations:** 1 Pneumologie Pédiatrique, Necker, AP-HP, Paris, France, Centre de Référence des Maladies Respiratoires Rares, Paris, France; 2 Réanimation pédiatrique, AP-HP, Bicêtre, France; 3 Pneumologie Pédiatrique, CHRU, Lille, France; 4 Chirurgie Pédiatrique, Necker, AP-HP, Paris, France; 5 Gynécologie Obstétrique, Antoine Béclère, AP-HP, Paris, France, Centre de Référence des Hernies Diaphragmatiques, Clamart, France; 6 Université Paris-Descartes, Paris, France; Centre Hospitalier Universitaire Vaudois, FRANCE

## Abstract

**Rationale:**

Congenital diaphragmatic hernia (CDH) is associated with a high incidence of respiratory problems, even after initial hospital discharge. These problems are likely to lead to re-hospitalization during infancy, although actual frequency of readmissions is unknown.

**Objective:**

We aimed to determine the rate of hospitalization for wheezing in infants with CDH between the time of initial discharge and 24 months of age, and to identify factors associated with readmission.

**Methods:**

Data about infants with CDH born in three French reference tertiary centers between January 2009 and March 2013 who were alive at hospital discharge, were extracted from a prospective national database.

**Results:**

Ninety-two children were identified, and 86 were included in the analysis. In total, 116 wheezing episodes requiring a doctor’s visit occurred in 50 infants (58%) before 24 months of age. Twenty-two children (26%) were readmitted at least once for wheezing exacerbations. RSV was present in 6 of 15 (40%) of children with available nasal samples at first readmission, and 1 of 5 (20%) at second readmission. Thoracic herniation of the liver, low gestational age, longer initial hospitalization, need for oxygen therapy at home, and eczema were all significantly associated with readmission for wheezing exacerbations. Fifty-three infants (62%) received palivizumab prophylaxis, but there was no association with the overall rate of readmission for wheezing exacerbations or RSV-related hospitalization.

**Conclusions:**

The rate of readmission for wheezing among infants with CDH is high, and significantly influenced by several prenatal and neonatal factors. Palivizumab prophylaxis was not associated with the rate of readmission.

## Introduction

Congenital diaphragmatic hernia (CDH) is a rare congenital malformation of the lung associated with high neonatal mortality [1]. Survivors of CDH have a high incidence of respiratory, nutritional, musculoskeletal, neurological and gastrointestinal problems, even after the initial period of hospitalization [1]. These problems are likely to lead to re-hospitalization during infancy, but the actual frequency of readmission is unknown. In particular, the risk of readmission for wheezing exacerbations has not been described. Several factors contribute to the poor respiratory health of survivors of CDH. Total alveolar number is reduced in these infants, mainly because of a definite reduced number of airway branches and hence of the number of acini [2]. In one series, up to 16% of them still require supplemental oxygen at the time of discharge for a mean duration of 14.5 months [3]. Furthermore, expiratory flows are lower in these infants than in healthy age-matched children [4], which increases the risk of early wheezing [5]. Small monocentric studies have reported that the rate of obstructive airway disease is high among both infants [3] and preschool survivors of CDH[6]. Respiratory syncytial virus (RSV) infection was also suggested to be a risk factor for the deterioration of respiratory health in survivors of severe CDH [7]. Consequently, the inclusion of prophylaxis with palivizumab in guidelines for the treatment of infants with CDH has been frequently discussed [8, 9]. A better understanding of the actual risk of readmission for respiratory causes in survivors of CDH, and the factors predicting hospitalization, is essential to define the best management policies for these children. Here, we used a national database of children with CDH in France to determine the rate of hospitalization for wheezing between initial discharge and 24 months of age. We also evaluated factors associated with readmission, including palivizumab prophylaxis.

## Methods

### Study design

A common database (Respirare) was established in France between several reference centers for the care of children with different rare respiratory disease in children, including CDH. Data about liveborn children with CDH are prospectively entered into the database via a secure Internet Protocol and web interface. The Respirare database is used as the electronic medical record for children routine visits, after initial hospital discharge. At each visit, to the rhythm set by the physician in charge of the child, the data are collected prospectively, according to predefined standardized items. Most authors of this study are physicians in charge of these children, and were those who collected the data. Retrospective analysis of data prospectively collected as part of routine visits is qualified as observational research by French law, as it does not involve any change in the management of patients, and no additional procedure for diagnostic or monitoring. In agreement with the French law for this type of research, parents received written information on possible use for research purposes of data collected during routine visits of their child, and on their right to oppose to such use. In the absence of opposition, a written agreement is not required. When extracting data for a clinical study, anonymisation was performed by a data manager who did not participate to the analysis. The authors responsible for the analysis had at their disposal only the initial names and date of birth. The database and data collection methods were approved by the French national data protection authorities, the “Commission Nationale de l’Informatique et des Libertés” and the “Comité Consultatif sur le Traitement de l'Information en matière de Recherche dans le domaine de la Santé”. Data quality is ensured by the users entering the data. A scientific committee meets twice a year to potentially improve the data management. This study was approved by the Institutional Review Board of the French Society for Respiratory Medicine, who confirmed its observational nature (CEPRO 2015–008).

Respirare was implemented gradually, with three centers (Paris-Necker, Clamart-Bicetre, and Lille) participating first. We identified patients born in these centers between January 2009 and March 2013 who were diagnosed with CDH either prenatally or immediately after birth, and alive at hospital discharge. All of the analyzed parameters were usually entered into the database during the initial hospitalization (prenatal and neonatal parameters, up to the initial discharge), and when the child comes to a routine clinic visit at the reference center for rare respiratory diseases in children. The intervals between clinic visits varied for each child, but all the children have at least two annual visits. Informations referring to items present in Respirare, but that have not been collected during a routine visit, were completed by a parental recall within 12 months after the last visit of the child. The missing data requested to the parents concerned either the clinical events after the initial discharge, or the child's environment. We did not collect more data than originally planned in Respirare. These data were integrated into the database. Ninety-two patients were identified from these centers.

### Data collection

Data concerning the prenatal parameters, the neonatal period, and the clinical events up to 2 years of age were collected. The term “infancy” was used in its largest definition, between the neonatal period and 2 years of age. The following data were collected: side of the CDH, fetal lung area to head circumference ratio (LHR), intra-thoracic localization of the liver, total fetal lung volume determined by magnetic resonance imaging (MRI), associated chromosomal abnormality, site of birth, gestational age at delivery, birth weight, length of stay in the intensive care unit (ICU), length of hospital stay, therapy administered at home (oxygen, enteral nutrition, inhaled corticosteroids, sildenafil, palivizumab prophylaxis), home environment (number of siblings, father and/or mother smoking (declarative), day-care attendance), father and/or mother allergic asthma or rhinitis, personal eczema, doctor’s visits for wheezing and hospitalization for wheezing and causes other than wheezing exacerbation. “Doctor’s diagnosis of recurrent wheezing” was defined by at least two wheezing episodes mentioned by a practitioner in the personal child health record.

Palivizumab is recommended in France (i) for children aged less than 6 months at the beginning of the epidemic period, and born at a term not exceeding 32 weeks; (ii) for children under 2 years old at the beginning of the epidemic period, born at a term not exceeding 32 weeks, and having a bronchopulmonary dysplasia which still required treatment within the last 6 months; and (iii) for children under two years of age with hemodynamically significant congenital heart disease. To these indications, the French Society of Neonatology has proposed adding children with underlying disease exposing them to prolonged hospitalizations in case of RSV infection, including infants with CDH. However, there is no clear consensus for this attitude, and palivizumab prevention was not proposed in the National treatment protocol established for children with CDH by the French High Authority of Health in 2012. To analyze the actual indications in our population, we considered that the period at risk of RSV infection began October 1 in children discharged from hospital before that date, or the day of their discharge from hospital for those who returned at home between October 1 and March 1.

### Statistical analysis

Data are presented as the mean ± standard error of the mean (SEM). Children who were not readmitted for wheezing were compared with those who were. Analysis of variance was used to compare continuous variables between groups. Qualitative variables were expressed as percentages, and chi-squared tests were used for comparisons. *P <* 0.05 was considered statistically significant. Analysis were also performed after adjustment on gestational age, with calculation of odds ratio (OR) and 95% confidence interval (CI). The rate of hospitalization and hospital stays in our population were compared with French epidemiological data for children less than 1 year of age in the general population in 2009 [10]. In this survey conducted by the French Institute for Public Health Surveillance, the hospitalization rate for wheezing in this age group was estimated at 35.8 / 1000 infants and the average length of stay was 4.3 days.

## Results

**Fig 1:**
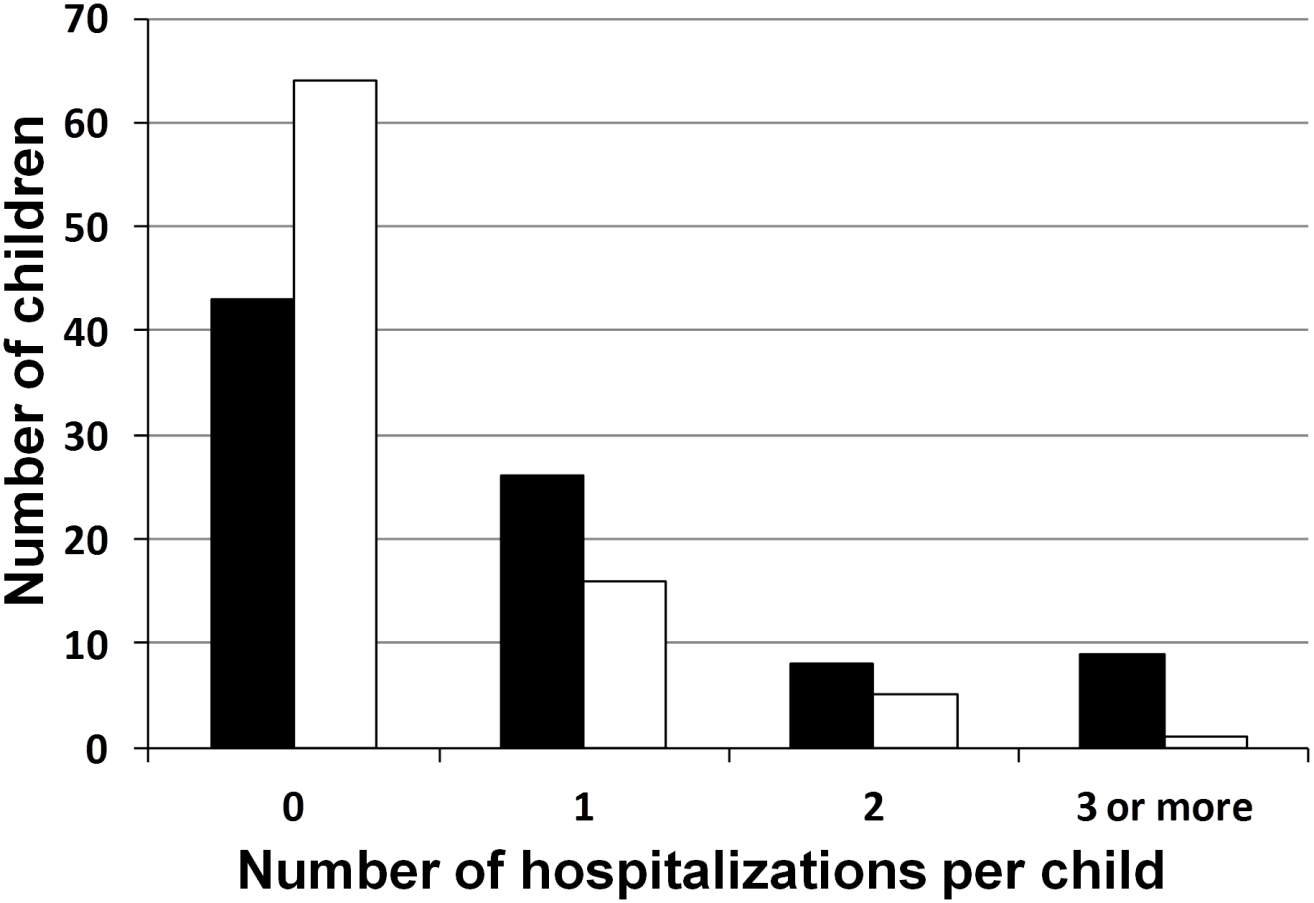
Readmissions among children with CDH, between hospital discharge and 24 months of age. The number of children who were readmitted zero, one, two, or three or more times is shown. Black columns correspond to hospitalizations for any cause, and white columns correspond to hospitalizations for wheezing exacerbations. The total number of children evaluated is n = 86.

Overall, we identified 92 children with CDH who were discharged from one of three reference centers. Six children were excluded from the analysis, because we were unable to contact the family to complete the data. The remaining 86 children were all still alive at 24 months. In total, 116 wheezing episodes requiring a doctor’s visit occurred in 50 infants (58%) before 24 months of age. Forty-three children (50%) were readmitted to hospital at least once between initial discharge and 24 months of age, including 22 (26%) for wheezing exacerbations (Fig 1). In total, 74 readmissions were recorded for these 43 children, including 31 readmissions for wheezing exacerbations. Non-respiratory causes for readmissions were: intestinal adhesion obstruction (n = 13), acute gastroenteritis (n = 7), other causes of infection (n = 8), feeding difficulties (n = 6), cardiac surgery (n = 2), pulmonary hypertension (n = 1), recurrence of hernia (n = 1), ablation of prosthetic patch (n = 1) and other non-respiratory problems (n = 4).

**Fig 2:**
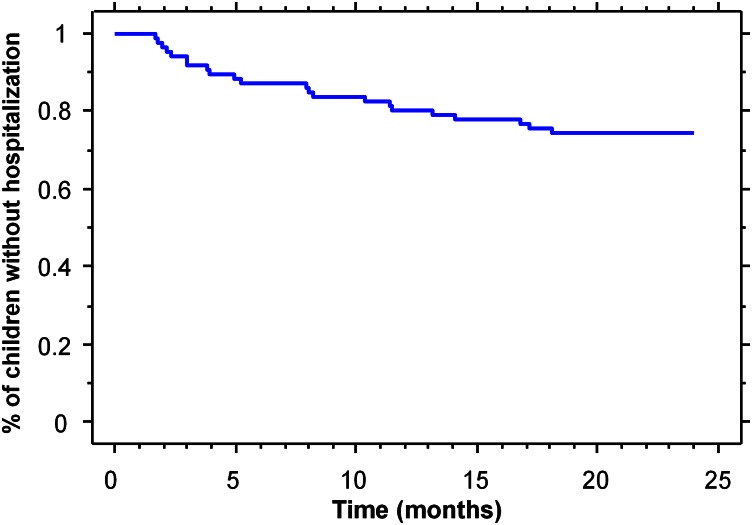
Kaplan—Meier analysis to estimate freedom from re-hospitalization.

Among the 22 children who were readmitted at least once for wheezing exacerbations, 77% of them were less than 12 months of age at the time of the first readmission (Fig 2). Five children were readmitted twice for wheezing before 24 months, and a child was readmitted five times during infancy. Table 1 summarizes the characteristics of the first and second readmissions. Overall, 8/22 (36%) of children required supplemental oxygen during the first readmission, and 3/6 (50%) during the second readmission. Children were discharged from hospital only after about 7 days; therefore, the length of stay was longer than the average of four days observed for children under 1 year of age with bronchiolitis in the general population [10]. Nasal samples were available for the analysis of RSV for 20 of the 31 hospitalizations. RSV was present in 6/15 (40%) of cases at first readmission, and in 1/5 (20%) at second readmission. No infant was hospitalized twice with an RSV infection.

**Table 1:** Characteristics of first and second readmissions for wheezing. The total number of children analyzed in each subgroup is specified if it differs from the total number available.

	First readmission (n = 22)	Second readmission (n = 6)
Age (months)	7.7 ± 1.2	10.6 ± 2.7
ICU admission, n (%)	2 (9)	1 (17)
Oxygen supply, n (%)	8 (36)	3 (50)
Mechanical ventilation, n (%)	1 (5)	1 (17)
Length of hospitalization (days)	7.0 ± 1.4	6.4 ± 2.0
RSV positive, n/N (%)	6/15 (40)	1/5 (20)

Several factors were associated with readmission for wheezing, including thoracic herniation of the liver, low gestational age, long initial hospitalization, need for oxygen therapy at home, and eczema (Table 2). When analysis was adjusted to gestational age, intrathoracic position of the liver, need for oxygen therapy at home, and eczema were still significantly associated with readmission for wheezing: OR (95% CI, p value) were 4.3 (1.2–14.8, *p*<0.03), 16.1 (1.6–166.7, *p*<0.02), and 4.9 (1.5–16.3, *p*<0.01), respectively. Children with a right-sided diaphragmatic hernia or those with a history of familial atopy also tended to be admitted more frequently to hospital, although the difference did not reach significance (*p* = 0.06 and *p* = 0.08, respectively). Among children who were not readmitted, 28 (44%) experienced at least one episode of wheezing requiring a doctor’s visit, and 17 (27%) had recurrent episodes of wheezing requiring a doctor’s visit. At two years of age, doctor’s diagnosed recurrent wheezing was significantly more frequent in infants who have been readmitted at least once (15/22, 68%) than in non-readmitted infants (17/64, 27%). Regular use of inhaled corticosteroids was present in 13/22 (59%) infants who have been readmitted at least once, and in 21/60 (35%) non-readmitted infants (p < 0.05).

**Table 2:** Characteristics of infants according to the occurrence of readmission for wheezing. Total number of children analyzed in each subgroup is specified, if different from the total number available.

	Infants who were readmitted for wheezing exacerbations (n = 22)	Infants who were not readmitted for wheezing exacerbations (n = 64)	p value
Left-sided hernia, n (%)	17 (77)	59 (92)	0.060
Abnormal karyotype, n/N (%)	1/17 (6)	3/53 (6)	1
LHR	1.5 ± 0.1	1.8 ± 0.1	0.135
MRI lung volume, o/e	0.37 ± 0.02	0.43 ± 0.01	0.113
Intrathoracic liver, n/N (%)	8/15 (53)	11/53 (21)	0.017
Inborn, n (%)	19 (86)	53 (83)	0.956
Gestational age (wks)	37.8 ± 0.6	39.3 ± 0.2	0.004
Birth weight (g)	2990 ± 180	3179 ± 64	0.213
ECMO, n	0	0	-
Prosthetic patch repair, n/N (%)	3/22 (14)	8/58 (14)	1
Length of ICU stay (d)	31.9 ± 6.8	20.3 ± 2.5	0.049
Length of hospital stay (d)	59.2 ± 10.9	35.8 ± 3.7	0.010
Home oxygen therapy, n (%)	4 (18)	1 (2)	0.019
Home enteral nutrition, n (%)	6 (27)	9 (14)	0.159
Pulmonary hypertension at discharge, n/N (%)	6/22 (27)	13/63 (21)	0.520
Older siblings at home, n/N (%)	13/21 (62)	34/63 (54)	0.526
Day-care attendance	5/22 (23)	20/62 (32)	0.531
Passive smoking (%)	4/20 (20)	18/61 (30)	0.587
Familial atopy (%)	13 (59)	24 (37)	0.078
Eczema (%)	9/20 (45)	10/61 (16)	0.012
Palivizumab prevention (%)	14 (64)	39 (61)	0.822

Fifty-three infants (62%) received palivizumab prophylaxis. There was no difference in age at the beginning of the risk period between children with prophylaxis and those without prophylaxis: 3.3 ± 0.4 months and 3.3 ± 0.5 months, respectively. All infants who received prophylaxis were aged less than 12 months at the beginning of the at-risk period, and 81% were aged less than 6 months. Monthly injections actually covered the whole at-risk period, at least until March 1, in 87% of infants for whom prophylaxis was initiated. Some parameters significantly associated with the risk of readmission have also influenced the prescription of palivizumab by practitioners. Children with palivizumab prophylaxis had a significantly longer initial hospitalization than those without prophylaxis: 50.3 ± 5.9 versus 27.2 ± 3.4 days, respectively (p < 0.005). Prematurity was also associated with a high rate of prophylaxis, although the difference was not significant with term infants: 7 of 8 premature infants (87.5%), with gestational age below 37 weeks, received prophylaxis, as compared to 45 of 77 term infants (58.4%) (not significant). Palivizumab was not associated with the rate of readmission for wheezing exacerbations (Table 2). Unadjusted odds ratio was 1.12 (95% CI: 0.41–3.06). Results were not modified when analysis was adjusted for length of hospital stay and gestational age: adjusted OR = 0.65 (95% CI: 0.21–2.03). Among children who were readmitted despite palivizumab prophylaxis (n = 14), all except one had received at least two injections before readmission. If we exclude from analysis the child having received only one injection before readmission, results are unchanged. In the analysis restricted to readmitted children with an available nasal sample (n = 20), readmission was related to the presence of RSV in 5 out of the 12 (42%) infants who received at least two monthly injections of palivizumab, and only 2 of the 8 (25%) infants who did not receive palivizumab prophylaxis.

## Discussion

Although children with CDH are considered at high risk of respiratory morbidity in the short and medium term after hospital discharge, few studies have evaluated the extent of this risk. In particular, the risk of readmission for wheezing exacerbations is not known. Our study is therefore the first to quantify this risk, and to identify the main predictors of readmission for wheezing.

An important advantage of our study is its multicentric design, which involved the use of a national database to incorporate prospectively data from each case. This method enabled us to assess the risk of respiratory complications in an unbiased population. Our study has however several limitations. Respirare brings the great advantage of a prospective multicenter collection of data, but at the cost of the pre-established selection of a limited number of data. The collection of these data is dependent on physicians at each routine visit of the child to the Reference Center, and is not based on a validated questionnaire. Finally, there are small number of outcomes in some subgroup analysis, limiting the power of the study.

The main finding of our study is that the rate of readmission for wheezing among infants with CDH is high. Overall, 26% of infants with CDH were readmitted for wheezing, mostly before 1 year of age, whereas this rate is only 3.6% for children of the same age in the French general population [10]. The rate of readmission for wheezing before 12 months of age was 18%, very close to the 13% rate of readmission for respiratory distress recently reported by Cauley et al. [6].

Abnormal lung development appear to be important risk factors of readmission for wheezing in infants with CDH. Several prenatal markers of lung volume, such as LHR, intrathoracic herniation of the liver, or total fetal lung volume determined by MRI are associated with postnatal survival [11, 12]. Low fetal lung volume also predicts neonatal morbidity, including the need for prosthetic patch repair, the duration of assisted ventilation and supplemental oxygen, and the incidence of feeding problems [13]. Among these markers, we found that only the position of the liver was significantly correlated with readmission for wheezing. Although the LHR and lung volume on MRI were smaller in infants who were readmitted than in those who were not, this difference was not significant. The limited power of our study may explain this lack of significance. By contrast, neonatal morbidity, which is itself favored by a small fetal lung volume, appeared to predict readmission for wheezing during infancy. The length of ICU and hospital stay, and sustained requirement for supplemental oxygen were all significantly associated with readmission. This finding is consistent with previous reports that use of pulmonary support on day 30 after birth is significantly associated with pulmonary and developmental morbidities at 1 and 5 years of age [14].

We also identified low gestational age as a risk factor for readmission in infants with CDH. Preterm birth is a well-recognized risk factor for bronchiolitis, and readmission for respiratory exacerbations during infancy [15], and also significantly worsens the short-term prognosis of CDH [16, 17]. Our study adds that among perinatal factors, preterm birth is not the only risk factor for wheezing in infants with CDH. Indeed, when adjusting analysis on gestational age, intrathoracic position of the liver and a long term requirement for supplemental oxygen were still identified as independent risk factors of readmission for wheezing.

In addition to the factors that are directly linked to CDH and its postnatal care, individual susceptibility factors may strongly contribute to the risk of readmission for wheezing. Indeed, eczema was significantly associated with readmission, independently of gestational age, suggesting a role for personal atopy. Moreover, recurrent wheezing, suggesting airway hyperreactivity, occurred significantly more frequently in infants with CDH who were readmitted for wheezing at least once than in those who were not readmitted to hospital. Long-term follow-up studies have shown that in survivors of CDH, the airways continue to present an obstructive pattern and are highly responsive to methacholine [1]. Infants and pre-school children with CDH are especially prone to these symptoms related to airway hyperreactivity, because the probability that children with CDH respond strongly to the inhalation of metacholine correlates negatively with age [18].

Finally, we found that palivizumab prophylaxis was not associated with the rate of readmission for wheezing. Palivizumab is very effective at preventing hospitalizations related to RSV in preterm infants [19, 20], as well as hospitalizations in infants with hemodynamically significant congenital heart disease [21]. Interestingly, RSV strongly influences the overall rate of hospitalization in these populations, because a reduction in RSV-related hospitalizations is associated with a significant reduction in the overall rate of hospitalization [20, 21]. In our study, palivizumab prophylaxis was not uniform, because of the absence of clear recommendation for such prophylaxis in infants with CDH. Nevertheless, there was no age-related bias in the evaluation of the association between palivizumab prophylaxis and risk for readmission for wheezing, and there was a good adequacy between actual palivizumab administration and at-risk period in infants for whom prophylaxis was initiated. Longer initial hospitalization and lower gestational age influenced the rate of prophylaxis, but there was still no significant association between palivizumab and readmission for wheezing exacerbations when analysis was adjusted for these two parameters. The absence of any relationship between palivizumab prophylaxis and readmission for wheezing suggests that RSV-related exacerbations do not significantly contribute to wheezing exacerbations in these infants. Furthermore, when we restricted the analysis to children with available nasal samples, palivizumab still had no effect on the rate of readmissions associated with RSV. Although this result is limited by the small number of children with available nasal samples, it raises doubt about the direct role of RSV in wheezing exacerbations in infants with CDH, and the rationale for providing palivizumab prophylaxis to these infants. Only a prospective multicentric randomised trial would be able to formally conclude on usefulness for palivizumab prophylaxis in this population.

## Abbreviations

CDH: Congenital diaphragmatic hernia
ICU: Intensive care unit
LHR: Lung area to head circumference ratio
MRI: Magnetic resonance imaging
RSV: Respiratory syncytial virus
